# Supplementary material for: A qualitative study examining the health system’s response to COVID-19 in Sierra Leone
Source: PLoS One. 2024 Feb 2;19(2):e0294391. doi: 10.1371/journal.pone.0294391 (PMC10836672; doi:10.1371/journal.pone.0294391)
Supplement: S1 Checklist — This questionnaire provides further information on the ethical, cultural, and scientific considerations specific to inclusivity in global research as they relate to this study. (DOCX) [file pone.0294391.s001.docx]

Inclusivity in global research

PLOS’ policy on inclusivity in global research aims to improve transparency in the reporting of research performed outside of researchers’ own country or community and ensures that PLOS publications reporting global research adhere to high standards for research ethics and authorship. Authors of relevant research articles may be asked to complete the questionnaire below, which outlines ethical, cultural, and scientific considerations specific to inclusivity in global research. This questionnaire may be requested when researchers have travelled to a different country to conduct research, if research uses samples collected in another country, research with Indigenous populations or their lands, or if research is on cultural artefacts. Researchers travelling to another country solely to use laboratory equipment will not normally be required to complete the questionnaire. However, the questionnaire can be requested at the journal’s discretion for any submission – if you have been requested to complete this questionnaire by the PLOS journal you submitted to, please do so.

Please complete the questionnaire below and include this as a Supporting Information file with your manuscript. Note that if your paper is accepted for publication, this checklist will be published with your article in the supporting information files. Please ensure that you reference the checklist in the main body of your manuscript. We suggest adding a subsection ‘Inclusivity in global research’ to your Methods section and adding the following sentence: “Additional information regarding the ethical, cultural, and scientific considerations specific to inclusivity in global research is included in the Supporting Information (SX Checklist)”

The questions have been designed to be applicable to a wide range of study types, and there are subsections for both human subjects research and non-human subjects research. If any of the questions are not relevant to your research please mark them as “N/A” as appropriate.

**Ethical considerations, permits and authorship**

*This section is applicable to all research types.*

Provide details as to who granted permissions and/or consent for the study to take place in the Methods section of your manuscript. This should include the names of **all** ethics boards, governmental organizations, community leaders or other bodies that provided approval for the study. If individuals provided approval refer to these people by their role or title but do not list their name(s).

Reported on page number: 8

If there were any deviations from the study protocol after approval was obtained please provide details of these changes in the Methods section of your manuscript.
Did this study involve local collaborators that are residents of the country where the research was conducted or members of the community studied? If you do not have any authors from said communities, please provide an explanation for this below.

Reported on page number: N/A

The paper has four co-authors that are residents of Sierra Leone. Two of them are nationals of Sierra Leone and two were residents in Sierra Leone at the time of the study but are not nationals of Sierra Leone.

Everyone listed as an author should meet PLOS’ criteria for authorship and all individuals who meet these criteria should be included in the author byline, rather than the acknowledgements. For further information please see the journal’s Authorship Policy.

**Human subjects research (e.g. health research, medical research, cross-cultural psychology)**

Did you obtain written informed consent from a representative of the local community or region before the research took place? How did you establish who speaks for the community? Details of written informed consent obtained from study participants should be reported separately in the Methods section of your manuscript.

The national authority, the Sierra Leone Ethics and Scientific Review Committee, reviewed the study and granted permission to undertake the study. This process involved submitting letters of support from the Ministry of Health and Sanitation and the Ministry of Defence. We did not seek broader written informed consent from community or regional representatives as the interviews targeted individual professionals and patients within healthcare settings at various locations who were willing and able to provide individual written informed consent. We collaborated with lead professionals at the healthcare facilities who acted as gatekeepers to interviewees, this took the form of formal discussion, written consent from gatekeepers was not requested.

How did members of the local community provide input on the aims of the research investigation, its methodology, and its anticipated outcome(s)?

Two of the local authors are also the Principle Investigators for this study, and were part of the research team from the inception of the study and remained involved through all stages, contributing to the study design, aims, methodology, the analysis of findings and their presentation for publication. Two addional authors were resident in Sierra Leone and were working in the health systems when the study was conducted. They were also part of the research team and contributed to all stages of the study.

When engaging with the local community, how did you ensure that the informed consent documents and other materials could be understood by local stakeholders?

The interviews were conducted by experienced and trained Research Assistants (RAs). Before signing up to take part in the study, participants had a discussion with the RAs about the purpose of the study, how it would be conducted and their commitment if they agreed to be interviewed. These discussions were held in the participants preferred language (Krio or English). Further discussion was held to go through and explain the consent documents and participants had the opportunity to ask questions, raise any concerns and the RAs were able to check their understanding. At all stages, participants were able to decline to take part if they so wished. They were also able to decline answering any question or to terminate the interview. Only one interview was conducted by phone on participant’s preference, the rest were conducted in person. All participants interviewed provided written signed informed consent. The person interviewed on phone had emailed the signed informed consent prior to the interview.

Will the findings of the research be made available in an understandable format to stakeholders in the community where the study was conducted (e.g. via a presentation, summary report, copies of publications, etc.)? Please provide details of how this will be achieved.

Hard copies of the publication will be made available centrally. Links to the online open access publication will also be shared with local/national stakeholders for self download via email and social media messaging platforms. Co-authors based at the local universities, ministries and health facilities will disserminate the paper and its findings in their respective organisations and networks.

**Non-human subjects research using specimens/ animals collected as part of the study, or those housed in archival collections. Examples include archaeology, paleontology, botany and zoology.**

Did the permission you obtained from a local authority to perform the study include an agreement on access to outputs and benefit sharing? This may include procedures to enable fair distribution of the benefits and resources arising from the research performed. Please include any details of Prior Informed Consent and Benefit Sharing Agreements obtained. These may be required by field-specific regulations, for example the Convention on Biological Diversity (CBD) and the associated Nagoya Protocol.

N/A

If the material used in your study was imported, please A) provide the year it was imported and B) indicate whether permits were obtained to import/export the materials used, C) provide details of any permits obtained. If this information is not available, please indicate this.

N/A

If you used archival specimens, please state how the material used in your study was acquired by the institute it is held in and provide details of any permits obtained for the original excavations/ sample collection. If this information is not available, please indicate this.

N/A

How was the potential cultural significance of the materials collected in your study to local communities considered in your research design? Were Indigenous peoples and/or local researchers and institutions involved with archaeological excavations / collection of specimens? If so, please provide a description of their involvement.

N/A

If your manuscript includes photographs of human remains please indicate whether authors obtained permission from descendants or affiliated cultural communities to do so.

N/A
