# Supplementary material for: A qualitative study examining the health system’s response to COVID-19 in Sierra Leone
Source: PLoS One. 2024 Feb 2;19(2):e0294391. doi: 10.1371/journal.pone.0294391 (PMC10836672; doi:10.1371/journal.pone.0294391)
Supplement: S1 File — (PDF) [file pone.0294391.s002.pdf]

Interview Guide – health staff

Section 1: Introduction

a. **To begin, can you describe your health facility?**
Probes:
- *Types of health facility (first-line, secondary, tertiary/referral care)*
- *Does it offer surgeries – emergencies and elective*

b. **What is your role in the health facility?**
Probes:
- *How long have you worked in the health sector and specifically in this facility?*
- *What is your position and responsibilities?*

c. **I am now going to ask a few questions on Corona virus.**
Can you please describe your knowledge of the Covid-19 outbreak? How is the covid-19 outbreak in your area?
Probes:
- *The extent/gravity of the outbreak?*
- *Do you know someone who has been tested positive for Covid-19? Were they close family/friend/neighbour?*
- *What can you tell me about Coronavirus – signs/symptoms, causes, transmission, prevention (social distancing), prognosis?*

d. **Has your role and responsibilities changed because of the Covid-19 outbreak?**
Probes:
- *Has your role changed?*
- *Have you got additional responsibilities?*

Section 2: Covid-19 impact (your health facility)

a. **How has your health facility responded to the outbreak?**
Probes:
- *Organisation changes that have occurred as a result of the outbreak such as setting up of Covid-19 response committees, triage to identify potential Covid-19 positive patients, opening times?*
- *Are you treating confirmed or suspected Covid-19 patients? If yes, what changes has been made to help with this?*

b. **Has there been any change in your health facility because of the Covid-19 outbreak?**

In terms of services available?
- *Changes in types of services available – has any service been discontinued or reduced? Why?*
- *Changes in caseloads especially related to surgeries and maternal health services? Why do you think this has happened?*

In terms of health workforce?
Probes:

- *Changes in staffing – number of staff, type/category of staff? why?*
- *Any staff have been deployed to other facilities? How has this affected your health facility?*
- *Have any staff fallen sick?*
- *Has there been any change in staff training and how has that affected work?*
- *General morale and stress among the health staff, and whether they fear catching Covid-19?*
- *Any support provided to staff to deal with these changes?*
- *Have staff received any training to deal with Covid-19 suspected/confirmed cases?*

In terms of funding?
Probes:
- *Any changes to the finances available to the health facilities?*
- *Any increases in costs because of Covid-19?*

In terms of medical supplies including equipment and technology
Probes:
- *Impact on availability of drugs/medical supplies – any stockouts?*
- *Availability of PPE as appropriate to the type of health facility*

In terms of communication and availability of information
Probes:
- *Are you receiving regular, clear and timely updates on the covid-19 outbreak?*
- *What information/communication is not available?*
- *Are you clear about the risks and priorities of the health sector and how your health facility needs to respond?*

<u>If the health facility does not conduct surgeries, skip to e</u>

c. I am now going to ask you about surgical care.
Has this service changed because of the Covid-19 outbreak?
Probes:
- *What changes have occurred?*
- *Any changes with regard to emergency surgeries and elective surgeries?*

Any changes in services and caseloads?
Probes:
- *What changes have occurred?*
- *Have you seen changes in numbers of patients presenting with non-Covid conditions?*
- *How is your facility coping with this?*
- *Are there differences with regard to emergency and elective surgeries?*

Any changes in staffing?
Probes:
- *What changes have occurred?*
- *Were there any changes in staff numbers, staff absence, working hours, pay and training?*
- *How did these changes affect provision of emergency care?*
- *How was the morale of the staff – workload and motivation?*
- *Are there differences with regard to emergency and elective surgeries?*

Any changes in availability of drugs/medical supplies/equipment?

Probes:

- *What changes have occurred – has there been an increase/reduction in stockouts?*
- *How is your facility coping with this?*
- *Are there differences with regard to emergency and elective surgeries?*

Any changes in funds available for surgeries?

Probes:

- *What changes have occurred?*
- *If extra funds were received, do you know how these were used? If less funds, then how did the facility cope with this?*
- *Are there differences with regard to emergency and elective surgeries?*

Any changes from the patient's perspectives?

Probes:

- *What changes have occurred?*
- *Do you feel patients can access surgical care as before?*
- *What has changed from their perspectives – increased costs, transport difficulties, confidence/fear in accessing care, unavailability of service/staff?*
- *Do you think patients are receiving the same quality of care as before the Covid-19 outbreak?*
- *Are there differences with regard to emergency and elective surgery care?*

d. I am now going to ask you about non-surgical care
How has this service changed because of the Covid-19 outbreak?

Probes:

- *What changes have occurred?*

Any changes in services and caseloads?

Probes:

- *What changes have occurred? Have you seen changes in numbers of patients presenting with non-Covid conditions?*
- *How is your facility coping with this?*

Any changes in staffing?

Probes:

- *What changes have occurred?*
- *Were there any changes in staff numbers, staff absence, working hours, pay and training?*
- *How did these changes affect provision of non-surgical care?*
- *How was the morale of the staff – workload and motivation?*

Any changes in availability of drugs/medical supplies/equipment?

Probes:

- *What changes have occurred – has there been an increase/reduction in stockouts?*
- *How is your facility coping with this?*

Any changes in funds available for non-surgeries services?

Probes:

- *What changes have occurred?*
- *If extra funds were received, do you know how these were used? If less funds, then how did the facility cope with this?*

Any changes from the patient's perspectives?
Probes:
- *What changes have occurred?*
- *Do you feel patients can access non-surgical services as before?*
- *What has changed from their perspectives – increased costs, transport difficulties, confidence/fear in accessing care, unavailability of service/staff?*
- *Do you think patients are receiving the same quality of care as before the Covid-19 outbreak?*

e. **Reflecting on the overall situation in your health facility, do you think the situation is similar in other facilities like yours?**
Probes:
- *Why or why now?*
- *Can you give few examples?*

f. **Were you around during the Ebola outbreak? Do you think the situation in your health facility is similar/better/worse now as compared to the Ebola time?**
Probes:
- *Is your facility in a better/worse position this time as compared to the Ebola outbreak?*
- *Why do you think so?*
- *What do you think has helped or why things have been better/worse this time?*
- *Any recommendations to improve the functioning of your health facility?*

Section 3: Covid-19 impact (overall health systems)

a. **How do you feel the health sector in Sierra Leone, overall, has responded the Covid-19 outbreak?**
Probes:
- *How well or poor has been the response? why?*
- *If you were around during the Ebola outbreak, has it been better/worse this time? Why?*
- *Any recommendations to improve the overall way in which the health sector responded to Covid-19 outbreak?*

Thank you very much for your time and information. Do you have any final comments or questions?

Demographics of the respondent:

- Sex (don't need to ask)
- Age
- Health facility (don't need to ask)
- Position/role

Interview Guide – patient/caretaker

Section 1: Introduction

a. **To begin, can you tell me where you have come from?**
Probes:
- *Is it a rural/urban area?*
- *How far is it from this facility?*
- *How did you come here? (own car/ambulance/bus/taxi etc)*

b. **What is the purpose of your visit to the health facility?**
Probes:
- *What services are you seeking here? Any surgeries?*
- *Was it an emergency or had this surgery been planned before?*
- *Were you referred by any other health provider? Who?*

Section 2: Covid-19 impact (general)

a. **I am now going to ask a few questions on Corona virus and how the epidemic has affected your life.**
Have you heard about Covid-19/Coronavirus? What have you heard about Coronavirus? How is the covid-19 outbreak in your area?
Probes:
- *The extent/gravity of the outbreak?*
- *Do you know someone who has been tested positive for Covid-19? Were they close family/friend/neighbour?*
- *What can you tell me about Coronavirus – signs/symptoms, causes, transmission, prevention (social distancing), prognosis?*

b. **What measures has the govt taken to deal with the Corona virus epidemic?**
Probes
- *Lockdowns?*
- *Social distancing?*
- *Face masks?*
- *Closures of schools, businesses, etc*

c. **How has your life changed because of Coronavirus or because of the govt restrictions?**
Probes
- *Has your day-to-day life changed because of this? How?*
- *Any change in your employment, schooling of children?*
- *Availability of essential services, cost of groceries, transportation, etc?*

d. **What are some of the bigger challenges you and your neighbours are dealing with to protect against Covid-19?**
Probes:
- *How are your neighbours coping with these challenges?*
- *Can people travel, work and socialise freely or are there any restrictions?*

- *How is social distancing or self-isolation managed in your neighbourhood? Are people adhering to this? If not, why not?*

e. **How is the Covid-19 outbreak different to the Ebola outbreak especially in terms of protecting you and your family from getting sick?**
Probes:
- *Are there any differences in govt guidelines this time as compared to Ebola – such as lockdowns, social distancing, wearing masks etc*
- *Do you think adherence to govt guidelines is better this time as compared to Ebola or worse? Why do you think so?*
- *Has the Ebola outbreak helped you and your community being prepared for this outbreak – such as in following government instructions on social distancing?*

f. **How well you think the national government has responded to the Covid-19 outbreak?**
Probes:
- *Why do you think so? Can you give examples of what was done well or managed poorly?*
- *Do you think you have received clear and timely information on how the government is responding?*
- *Do you think the government is doing enough or do you feel they are probably doing too much? Why do you think so?*

Section 3: Access to health care

a. **I am now going to ask you a few questions on the care you received in this health facility? Why are you at the health facility today? Why did you decide to come to this facility?**
Probes:
- *Why did you come to this particular health facility?*
- *Were you referred from another health provider?*
- *Do you know a health staff here? Have you been to this facility before?*
- *Was it the cost of treatment, quality of care, transport and near to your home?*

b. **Did you face any difficulties receiving care for this problem because of the Covid-19 outbreak?**
Probes:
- *Any difficulties in transportation/travel? How did you solve this?*
- *Additional out-of-pocket expenses because of the Covid-19 outbreak? Where did you get the funds to cover these expenses?*
- *Unavailability of health staff, closures of other health facilities?*

c. **What has been your experience so far with the service at this facility?**
Probes:
- *Were there any delays in receiving health care? Why?*
- *Are you satisfied with the care you/patient has received so far?*
- *Do you feel it would have been different if there was no Covid-19 outbreak? In what way?*

d. **Do you think there is any difference in service provision and access between people with**

Covid-19 and people with other health care needs?
Probes:
- *Who in your opinion is more likely to receive care?*
- *Are there also differences in terms of provision, staff attitude, resources available at the health facility to treat Covid-19 patients vis-a-vis other patients?*
- *Do you agree with the additional resources assigned to Covid-19 care even if it's at the cost of non-COVID-19 care?*

e. **Do you have any recommendation on how the health facility can provide better care during a pandemic such as the Covid-19 outbreak?**

We are almost done. I will now ask for some basic information about you:

- Sex (don't need to ask)
- Age
- Highest education level/degree
- Employment
- Health facility (don't need to ask)
- Patient or Caretaker (don't need to ask)
- Type of health service sought

Thank you very much for your time and information. Do you have any final comments or questions?

Interview Guide – Key informants

Section 1: Respondent's organisation and role

a. **To begin, can you describe your organization and how it is involved in the Covid-19 planning?**
Probes:
- *What is the main purpose/work of your organization?*
- *Are you involved in the national/regional level planning for Covid-19? In which way?*

b. **What is your role in the organization? Has it changed because of the Covid-19 outbreak?**
Probes:
- *What is your position and responsibilities?*
- *How long have you worked in this position?*
- *Has your role and responsibilities changed during the COVID-19 outbreak? How?*

Section 2: Covid-19 impact on the health systems

a. **We are interested in understanding Sierra Leone's response to Covid-19 outbreak. How do you feel the health sector in Sierra Leone, overall, has responded the Covid-19 outbreak? Has it changed over time – from the beginning of the outbreak to now?**
Probes:
- *How well or poor has been the response? Why do you think so?*
- *Can you provide few examples?*

b. **As compared to the Ebola outbreak, has the response been better/worse this time? And in what way? Can you give examples?**
Probes:
- *Why do you think so?*
- *Can you give examples of what has worked better?*
- *Can you give examples of what has been worse?*

c. **Do you think there were any learnings from the Ebola outbreak that shaped the current response? Can you give examples?**
Probes:
- *What particular aspects were transferable to the Covid-19 outbreak?*
- *Can you give any examples? (PPE, disease control training, C-19 triage at hospitals, etc)*
- *Were there any missed opportunities – any aspects that should have been adopted for the Covid-19 planning but were not? Any examples?*

d. **Do you think there has been any effect on non-Covid-19 services in the health systems – especially emergency surgeries (such as caesarean sections), elective surgeries (such as hernia surgeries) and other maternal health services? Has this changed over time – from the beginning of the outbreak to now?**
Probes:
- *How has emergency care been impacted by Covid-19?*
- *Have elective surgeries been impacted by Covid-19?*
- *How has other services such as maternal health care been impacted?*

- *Has there been any policy changes regarding staff training or in training institutions because of Covis-19? What are these changes?*
- *Are/will there be any long-term effects to these services? Why?*

e. **What measures (if any) were taken to mitigate these impacts? From the beginning of the outbreak to now.**
   Probes:
   - *Any incentives or additional funding?*
   - *Changes in opening hours?*
   - *Changes in staff availability/training?*
   - *Designated C-19 treatment centres?*

f. **What has been the major challenges faced by the health systems in handling the Covid-19 outbreak? These could be in the leadership, managing health facilities and staff or even in terms of communicating with people. Did this change over time – from the beginning of the outbreak to now?**
   Probes:
   - *Shortages in medical supplies (PPE, oxygen, testing kits, etc)?*
   - *Any additional funding to the health systems?*
   - *Leadership and decision-making. Communication within the health system*
   - *Technical know-how*
   - *Role of international organisations such as the WHO*
   - *Communicating with the public*

g. **How is the health system coping with these challenges? What were some of the measures taken? Has this changed over time – from the beginning of the outbreak to now?**
   Probes:
   - *Additional funding from international donors*
   - *Financial and other incentives to health staff*
   - *Engaging with community organisations etc*

h. **Do you think the situation is similar to other countries like yours? Can you provide a few examples where Sierra Leone has done better and where other countries have done better? Why do you think so? Also, reflecting on the situation from the beginning of the outbreak to now?**
   Probes:
   - *Why do you think Sierra Leone has done better/worse than other countries?*

Section 3: Leadership and decision making

a. **We would like to understand the decision-making process around Sierra Leone's Covid-19 health system response. In the health sector, who all are involved in policy decisions? Did this change over time – from the beginning of the outbreak to now?**
   Probes:
   - *Have the govt set-up different task forces? How are they structured and what is their role?*
   - *Which ministries/directorates are involved? Membership of these task forces.*

b. **Are there other organisations or individuals, outside the government, who are playing a**

key role in decision making? Did this change over time – from the beginning of the outbreak to now?
Probes:
- *Who is advising the government/MOHS?*
- *Which organizations is the MOHS consulting with (WHO, iNGOs etc)?*

c. **In your opinion, how is this decision-making process going on? What is not working well and what can be improved and how? Did this change over time – from the beginning of the outbreak to now?**
Probes:
- *In your opinion, is the communication, coordination and partnership between these organisations going well?*
- *Are the decisions based on data/evidence? Can you give an example?*
- *Anyone/organisation left out and should be consulted when making policy decisions?*

Section 4: Future

a. **Thinking of the future and how the Covid crisis is unfolding, what do you think will be the main challenges for the health system in Sierra Leone?**
Probes:
- *Funding? Testing? Public support in curtailing the spread of the virus?*

b. **Do you have any recommendations on how Sierra Leone can prepare better for a future pandemic?**
Probes:
- *Increased funding to health system, more staff training on disease control, better surveillance system, etc*

Thank you very much for your time and information. Do you have any final comments or questions?

Interviewer to note the following:

- Respondent's Sex
- Respondent's Organization
- Respondent's Job title
