# Supplementary material for: A qualitative study examining the health system’s response to COVID-19 in Sierra Leone
Source: PLoS One. 2024 Feb 2;19(2):e0294391. doi: 10.1371/journal.pone.0294391 (PMC10836672; doi:10.1371/journal.pone.0294391)
Supplement: Attachment — Submitted filename: Reviewer Comments_13_08_2023.docx [file pone.0294391.s003.docx]

The authors have presented an impactful and interesting study entitled: “We plan because if you don’t plan, you plan to fail” - A qualitative study examining the health system’s response to COVID-19 in Sierra Leone”. The authors may consider addressing the following comments to help improve the quality of the papers.

(1) The title may be revised as it may not be informative or clear to most readers.
(2) The abstract should clearly show the backgrounds (including aims), methods, results, and conclusion.
(3) Detailed information about the recruitment/interview should be provided. E.g. :
(a) How did authors ensure that participants understood the interview questions ?
(b) At what stage did authors achieved a thematic saturation ? .
(c) How the transcripts were coded ? ,
(d) Potential disagreement in the coding of the transcript, and how resolved if any ?
(4) In Line 151: Data Analysis, the information provided are very general. Authors needs to explain in detailed how was data going to be analysed? Need more detail here.
