# Supplementary material for: A qualitative study examining the health system’s response to COVID-19 in Sierra Leone
Source: PLoS One. 2024 Feb 2;19(2):e0294391. doi: 10.1371/journal.pone.0294391 (PMC10836672; doi:10.1371/journal.pone.0294391)
Supplement: Attachment — Submitted filename: Response letter.docx [file pone.0294391.s004.docx]

**RESPONSE TO REVIEWERS’ COMMENTS**

We would like to thank the editor and the reviewers for taking the time to review this study. Their comments have helped us to strengthen the paper. Below, we have provided a point-by-point response to the comments.

Please note the location of revisions referred to below is as per the document showing track changes.

**JOURNAL REQUIREMENTS**

**When submitting your revision, we need you to address these additional requirements.**

**1. Please ensure that your manuscript meets PLOS ONE's style requirements, including those for file naming. The PLOS ONE style templates can be found at**

**https://journals.plos.org/plosone/s/file?id=wjVg/PLOSOne_formatting_sample_main_body.pdf and**

[**https://journals.plos.org/plosone/s/file?id=ba62/PLOSOne_formatting_sample_title_authors_affiliations.pdf**](https://journals.plos.org/plosone/s/file?id=ba62/PLOSOne_formatting_sample_title_authors_affiliations.pdf)**.**

**RESPONSE:** Style templates for both the main body text and authors’ affiliation have been reviewed and the manuscript formatting has been altered in line with this guidance. A supporting information section has been added at the end (page 42)

**2. Please provide additional details regarding participant consent. In the ethics statement in the Methods and online submission information, please ensure that you have specified what type you obtained (for instance, written or verbal, and if verbal, how it was documented and witnessed). If your study included minors, state whether you obtained consent from parents or guardians. If the need for consent was waived by the ethics committee, please include this information.**

**Once you have amended this/these statement(s) in the Methods section of the manuscript, please add the same text to the “Ethics Statement” field of the submission form (via “Edit Submission”).**

**For additional information about PLOS ONE ethical requirements for human subjects research, please refer to** [**http://journals.plos.org/plosone/s/submission-guidelines#loc-human-subjects-research**](http://journals.plos.org/plosone/s/submission-guidelines#loc-human-subjects-research)**.**

**RESPONSE:** Written signed informed consent was obtained from all participants before they could be interviewed.

**3. Please include a complete copy of PLOS’ questionnaire on inclusivity in global research in your revised manuscript. Our policy for research in this area aims to improve transparency in the reporting of research performed outside of researchers’ own country or community. The policy applies to researchers who have travelled to a different country to conduct research, research with Indigenous populations or their lands, and research on cultural artefacts. The questionnaire can also be requested at the journal’s discretion for any other submissions, even if these conditions are not met. Please find more information on the policy and a link to download a blank copy of the questionnaire here: https://journals.plos.org/plosone/s/best-practices-in-research-reporting. Please upload a completed version of your questionnaire as Supporting Information when you resubmit your manuscript.**

**RESPONSE**: We have completed the questionnaire. It is included as Supporting Information (S1) with the manuscript. As suggested in the guidance, we have also cross-referenced the inclusivity questionnaire in the methods section so readers are directed to this questionnaire for further information.

**4. Please provide the full name of the ethics committee from Sierra Leone that approved your study.**

**RESPONSE:** The ethics committee at Sierra Leone is:

Name: The The Sierra Leone Ethics and Scientific Review Committee (SLESRC).

Address: Government of Sierra Leone, Office of the Ethics and Scientific Review Committee, Ministry of Health and Sanitation, Directorate of Policy and Planning (DPPI), Youyi Building, Fifth Floor, East Wing, Sierra Leone.

The full name of the ethics committee is included in the manuscript on page 8.

**REVIEWER'S RESPONSES TO QUESTIONS**

**Comments to the Author**

**1. Is the manuscript technically sound, and do the data support the conclusions?**

**The manuscript must describe a technically sound piece of scientific research with data that supports the conclusions. Experiments must have been conducted rigorously, with appropriate controls, replication, and sample sizes. The conclusions must be drawn appropriately based on the data presented.**

**Reviewer #1: Partly**

**Reviewer #2: Yes**

**RESPONSE**: After making the revisions as suggested by the reviewers, we hope this comment is now addressed fully.

________________________________________

**2. Has the statistical analysis been performed appropriately and rigorously?**

**Reviewer #1: N/A**

**Reviewer #2: N/A**

________________________________________

**3. Have the authors made all data underlying the findings in their manuscript fully available?**

**The PLOS Data policy requires authors to make all data underlying the findings described in their manuscript fully available without restriction, with rare exception (please refer to the Data Availability Statement in the manuscript PDF file). The data should be provided as part of the manuscript or its supporting information, or deposited to a public repository. For example, in addition to summary statistics, the data points behind means, medians and variance measures should be available. If there are restrictions on publicly sharing data—e.g. participant privacy or use of data from a third party—those must be specified.**

**Reviewer #1: No**

**Reviewer #2: Yes**

**RESPONSE:** Our data included qualitative data/interviews with key stakeholders, health staff and patients in Sierra Leone. The data is available on request from the corresponding author but we are unable to share the data in a public space because we did not take permission from respondents in the consent forms to do so and for some transcripts (esp, key informants) anonymity can not be fully ensured.

________________________________________

**4. Is the manuscript presented in an intelligible fashion and written in standard English?**

**PLOS ONE does not copyedit accepted manuscripts, so the language in submitted articles must be clear, correct, and unambiguous. Any typographical or grammatical errors should be corrected at revision, so please note any specific errors here.**

**Reviewer #1: Yes**

**Reviewer #2: Yes**

________________________________________

**REVIEWER #1**

**The topic of the study is relevant and interesting. However, the paper needs some revision in some of the sections.**

**1. It would be interesting to know the rationale for choosing surgical and maternity care as an indicator of overall health system functioning as both types of health services are for special populations.**

**RESPONSE:** We focussed on surgical and maternity care as they cover both routine services and emergency care. During the Ebola epidemic, major surgeries and routine care were disruption more than emergency care such as caesarean deliveries. We have revised the paper accordingly:

(Introduction, last paragraph, pages 5-6): *“The Ebola epidemic caused severe disruptions in major surgeries and routine maternity care, while emergency care such as caesarean deliveries were more resilience. We, therefore, focus on the provision of emergency and routine care, specifically, maternal, and surgical services.”*

**2. The methodology section could be revised by including the following information:**

**a. How were the interview guidelines developed? Was it pilot tested on any participants?**

**b. How were the study participants approached? Was there a process to set up an appointment with the study participants for the interview?**

**c. How were the interviews with the study participants conducted? Was it in-person or via some virtual platforms like Zoom, WhatsApp, Teams?**

**d. How did the authors decide on the sample size of the study or how was the sample size deemed sufficient for the study?**

**e. Were the translated audios independently checked by the team members to ensure the reliability of the translation?**

**f. Please revise Table 2 as the numbers do not add up to a sample size of 24.**

**RESPONSE:** Thank you for these comments. We have added more information in the methods section and corrected the numbers in Table 2.

(Methods, Study design, pages 7-8): *“We interviewed health staff, patients, and key informants (KIs). Health staff were identified by the facility heads depending on their availability and experience of working in the surgical/maternity wards or those involved with the management of the facility. In each facility, at least two health staff and one adult patient were interviewed. Additional senior staff were interviewed from secondary and tertiary hospitals who has a more district-wise perspective. The patients were approached by the staff in maternity and surgical wards and if they agreed to be interviewed, they were later interviewed at their home or a convenient location. KIs were identified by the local research team. Two were involved in the national COVID-19 response holding national planning and policy roles and one was involved in providing technical support from an international organisation (Table 2).*

**Table 2. Interviewee demographics (n=24)**

| **Role** | **Male** | **Female** | **Total** |
| --- | --- | --- | --- |
| *Health workers* (n=16) | | | |
| Doctor | 2 | 1 | 3 |
| Surgeon | 3 | 0 | 3 |
| Midwife | 0 | 1 | 1 |
| Maternal & Child Health Aide | 0 | 1 | 1 |
| Nurse | 0 | 1 | 1 |
| Matron | 0 | 4 | 4 |
| Finance Officer  Deputy in charge | 0  1 | 2  0 | 2  1 |
| Patients (n=5) | 2 | 3 | 5 |
| Key informants (n=3) | 3 | 0 | 3 |

*Interviews were conducted by two experienced and trained Research Assistants (RAs). The RAs received a 2-day training consisting of mock interviews, transcription training and a pilot interview. The interviews were conducted in English or Krio, depending on participant preference, audio-recorded and transcribed by the RAs. Krio interviews were translated during transcription. A sample of transcriptions (30%) were crossed checked. The last author cross-checked the English transcriptions and the RAs checked each other’s Krio transcriptions. Twenty-three interviews were conducted in person; one KI interview was conducted by phone. Interviewers ensured understanding of the questions by repeating or rephrasing as needed. All respondents provided written signed informed consent.*

*The interview guide was based on the WHO’s Health System Building Blocks [17] and asked questions on changes to service provision, health staff, finances of health facilities, access to information and medical supplies, and leadership. Patients were additionally asked about experiences of accessing healthcare and KI responses provided organisational and national perspectives on COVID-19 health systems response. All respondents were asked to reflect on their experience and learnings from the Ebola epidemic. Interview guides were piloted in a health facility by the RAs as part of their training. Interview guides are included as supplementary files (S1 File).”*

**3. While the efforts of the authors to create Figure 1 is appreciable, Figure 1 can be decluttered to make it more visually effective.**

**a. Please add a note for any abbreviations used in the Figure.**

**RESPONSE:** Figure 1 has been redesigned to appear clearer and more impactful. Joining arrows have been replaced by colour coding and theme titles have been changed to a larger darker bold font. Oval theme sections have been resized to better fit the page and words within, a background shading has been used to help text stand out. A note the abbreviation has been added. We have included as a separate file as adding it to the main document was causing the picture resolution to reduce.

**REVIEWER #2:**

**The authors have presented an impactful and interesting study entitled: “We plan because if you don’t plan, you plan to fail” - A qualitative study examining the health system’s response to COVID-19 in Sierra Leone”. The authors may consider addressing the following comments to help improve the quality of the papers.**

**(1) The title may be revised as it may not be informative or clear to most readers.**

**RESPONSE:** The title has been simplified to convey the purpose and scope of the study succinctly to readers.

Line 2-3 *“A qualitative study examining the health system’s response to COVID-19 in Sierra Leone.”*

**(2) The abstract should clearly show the backgrounds (including aims), methods, results, and conclusion.**

**RESPONSE: We have identified the four sections below but because of the journal requirements, the sub-headings are not included in the submitted manuscript. The text in bold has been added to identify the aims and conclusion.**

*Background: The paper examines the health system’s response to COVID-19 in Sierra Leone. It* ***aims to*** *explore how the pandemic affected service delivery, health workers, patient access to services, leadership, and governance. It also examines to what extent the legacy of the 2013-16 Ebola outbreak influenced the COVID-19 response and public perception.*

*Methods: Using the WHO Health System Building Blocks Framework, we conducted a qualitative study in Sierra Leone where semi-structured interviews were conducted with health workers, policymakers, and patients between Oct-Dec 2020. We applied thematic analysis using both deductive and inductive approaches. Twelve themes emerged from the analysis: nine on the WHO building blocks, two on patients’ experiences, and one on Ebola.*

*Results: We found that routine services were impacted by enhanced infection prevention control measures. Health workers faced additional responsibilities and training needs. Communication and decision-making within facilities were reported to be coordinated and effective, although updates cascading from the national level to facilities were lacking. In contrast with previous health emergencies which were heavily influenced by international organisations, we found that the COVID-19 response was led by the national leadership. Experiences of Ebola resulted in less fear of COVID-19 and a greater understanding of public health measures. However, these measures also negatively affected patients' livelihoods and their willingness to visit facilities.*

*Conclusion:* ***We conclude,*** *it is important to address existing challenges in the health system such as resources that affect the capacity of health systems to respond to emergencies. Prioritising the well-being of health workers and the continued provision of essential routine health services is important. The socio-economic impact of public health measures on the population needs to be considered before measures are implemented.”*

**(3) Detailed information about the recruitment/interview should be provided. E.g. :**

**(a) How did authors ensure that participants understood the interview questions ?**

**(b) At what stage did authors achieved a thematic saturation ? .**

**(c) How the transcripts were coded ? ,**

**(d) Potential disagreement in the coding of the transcript, and how resolved if any ?**

**(4) In Line 151: Data Analysis, the information provided are very general. Authors needs to explain in detailed how was data going to be analysed? Need more detail here.**

**RESPONSE:** More information on the recruitment and conduction of interviews (3a) has been addressed to address comments from Reviewer #1 (comment 2). Please refer to our response there.

With regard to analysis (3b-d and 4), we have now included more information on the process.

(Methods - Data Analysis; page 9): *“**Transcripts were read multiple times for immersion before thematic analysis was conducted. We used both inductive and deductive approaches [21, 22] to identify themes and sub-themes. The deductive approach identified themes based on the WHO Health System framework [20] which matched the design of the interview questions. Additional themes about the patient experience and learning from Ebola, not captured in the WHO Health System framework, were identified inductively. Coding was conducted by HS using NVivo 12. Initial codes were reviewed and agreed by three authors (HS, EB and DP) and the final themes and subthemes were decided in consultation with other authors. Theme saturation was reached during data analysis when all transcripts had been read multiple times and coding completed. We believe if it were feasible to interview more patients and national response KIs, additional new themes outside of the thematic framework may have emerged providing alternative insights to those presented”.*
